# A synthetic multi-cellular network of coupled self-sustained oscillators

**DOI:** 10.1371/journal.pone.0180155

**Published:** 2017-06-29

**Authors:** Miguel Fernández-Niño, Daniel Giraldo, Judith Lucia Gomez-Porras, Ingo Dreyer, Andrés Fernando González Barrios, Catalina Arevalo-Ferro

**Affiliations:** 1 Departamento de Biología, Universidad Nacional de Colombia, Bogotá, Colombia; 2 Grupo de Diseño de Productos y Procesos (GDPP), Departamento de Ingeniería Química, Universidad de los Andes, Bogotá DC, Colombia; 3 Centro de Bioinformática y Simulación Molecular (CBSM), Universidad de Talca, Talca, Chile; 4 Heisenberg-Gruppe BPMPB, Universität Potsdam, Potsdam, Germany; Imperial College London, UNITED KINGDOM

## Abstract

Engineering artificial networks from modular components is a major challenge in synthetic biology. In the past years, single units, such as switches and oscillators, were successfully constructed and implemented. The effective integration of these parts into functional artificial self-regulated networks is currently on the verge of breakthrough. Here, we describe the design of a modular higher-order synthetic genetic network assembled from two independent self-sustained synthetic units: repressilators coupled via a modified quorum-sensing circuit. The isolated communication circuit and the network of coupled oscillators were analysed in mathematical modelling and experimental approaches. We monitored clustering of cells in groups of various sizes. Within each cluster of cells, cells oscillate synchronously, whereas the theoretical modelling predicts complete synchronization of the whole cellular population to be obtained approximately after 30 days. Our data suggest that self-regulated synchronization in biological systems can occur through an intermediate, long term clustering phase. The proposed artificial multicellular network provides a system framework for exploring how a given network generates a specific behaviour.

## Introduction

Designing multicellular systems to exhibit finely tuned coordinated behaviour is a major challenge in synthetic biology. In the past decade, the main efforts in this field focused on the study of simplified elements, such as switches[1–3], cascades [4], pulse generators [5], oscillators [6–10] and logic gates [11,12], for instance. All of these circuits usually aim at controlling isolated cellular functions. Right now, we are at the cusp of the second wave of synthetic biology, where the separate parts and modules need to be integrated to create a system level circuitry [13]. This significant step forward will be of enormous help to understand how various phenomena arise from the connectivity of genes and proteins. At the theoretical level several studies already addressed the problem of coupled synthetic units [14–16], as a means to study open biological questions.

However, only recently first experimental breakthrough was achieved in this context [17,18]. By combining elements from the quorum sensing machineries of *Vibrio fischeri* and *Bacillus thuringiensis* Danino and Co-workers engineered a systemic genetic clock in an *Escherichia coli* population. Global oscillations of the artificial quorum sensing module (placed in separate cells) were induced when a critical cell density was reached [17,18]. The timer in this global system is the concentration of *N*-(3-oxohexanoyl) homoserine lactone (AI), the signal transmitter in bacterial quorum sensing. Whereas the synthetic module does not produce self-sustained oscillations and does not oscillate in an individual cell, it only oscillates when a significant number of cells are present and the intercellular communication via AI induces bulk oscillations. Until now, every designed system relies on high concentrations of AI, while low concentrations do not induce expression in the system. For this reason, the circuit needs to be placed on a well where high cellular concentrations can be reached [18] Nonetheless, this is doubtlessly an important step towards the goal of synchronising autonomous, cell-intrinsic clocks.

In this study we go one step further. We combine two modular units to generate a complex, higher order network: one is the repressilator [6], an autonomous, self-sustained oscillator, and the other is an artificial cell-cell communication module constructed from entities of the quorum sensing systems of *V*. *fischeri* and *Agrobacterium tumefaciens*. The main advantage of this “plug-and-play” design of the network is the independence of both components—the oscillator and the communication circuit can be manipulated irrespective of each other. Moreover, although the proposed modular system is not a derivative of a natural network, it still offers the possibility to study specific functions and building principles for which limitations occur in the natural environment.

The repressilator can produce autonomous oscillations when in isolation [6], whereas the artificial cell-cell communication module allows the coupling between the repressilators of separate cells. This in turn results in a global enhancement of the oscillatory response of the system, as previous theoretical investigations have suggested [15]. However, the synthetic communication module which we present here needed to be significantly modified in comparison to their predecessors from theoretical work [15, 19, 20], in order to account for experimental difficulties as described further. Moreover, it is important to note that coupling among oscillators is not sufficient to achieve synchronization [21]. Our experimental and theoretical results suggest that self-regulated synchronization in biological systems can occur through an intermediate, long term clustering phase. The proposed synthetic network therefore provides a well-defined test bed for the analyses of the collective behaviour of a population of coupled oscillators.

## Results and discussion

### Network of coupled oscillators

**Fig 1:**
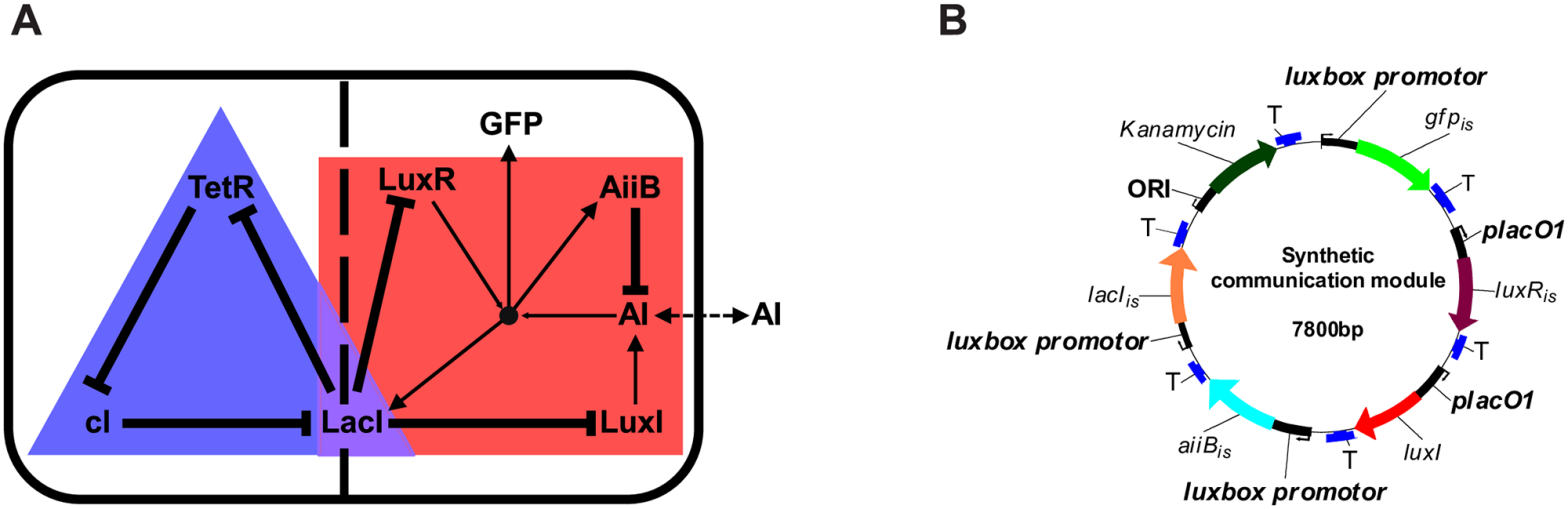
**(A) Model of the modular network containing two units**: the repressilator [6] (blue triangle), a network where three genes (*tetR*, *lacI*, *λcl*) suppress the expression of each other, and the artificial cell-cell communication module (red square). This artificial module was built from components of the quorum sensing systems of *Vibrio fisheri* and *Agrobacterium tumefaciens*. The LuxI protein synthesizes an Auto-inductor AI (3-oxo-C6HL). Upon reaching a critical concentration, AI is bound by the LuxR receptor; AI-LuxR binds to the *luxbox promoter* region and activates gene transcription. In the artificial cell-cell communication module, the AI-LuxR complex stimulates production of GFP (Green Fluorescent Protein), expression of the lactonase AiiB, and LacI, the repressor of *pLac01*. AiiB can hydrolyze AI molecules creating a negative feedback to restart the system. **(B)** Scheme of the recombinant plasmid coding for the artificial cell-cell communication module.

The network we present here (Fig 1a) is composed of two separate components: the repressilator [6] (Fig 1a, *blue shaded*), an autonomous, self-sustained oscillator, and an artificial communication module constructed from entities of the quorum sensing systems of *Vibrio fischeri* and *Agrobacterium tumefaciens* (Fig 1a, *red shaded*; Fig 1b). Initially, our experimental design was based on a coupling scheme proposed by Garcia-Ojalvo *et al*. (2004) including a slight modification (Figure A in S1 File, left panel): In particular, LuxR was placed under the control of LacI, in order to ensure adequate concentrations of the LuxR-AI complex, which acts as a transcription factor for the GFP promoter. We uncovered that if the LuxR is constitutively produced, any available AI concentration will lead to GFP expression. However, even the modified scheme produced difficulties. We observed an over-expression of GFP due to accumulation of the AI molecules in the media, hindering the observation of synchronous oscillations (Figure A in S1 File, right panel). To overcome this problem, we further added a step that controls the concentration of *N*-(3-oxohexanoyl) homoserine lactone molecules. We introduced in our network an acyl-HSL hydrolase (AiiB) from *Agrobacterium tumefaciens*, which degrades the autoinducer.

Within the communication module, LuxI is an autoinducer (AI) synthase from *V*. *fischeri* that catalyzes the production of the diffusible autoinducer *N*-(3-oxohexanoyl) homoserine lactone. LuxR is a transcriptional activator from *V*. *fischeri* that is active in the presence of the autoinducer. The activated LuxR binds to the *luxbox* promoter and induces the expression of GFP, AiiB and LacI. The green fluorescent protein GFP serves as reporter protein; AiiB, an acyl-HSL hydrolase from *Agrobacterium tumefaciens*, degrades the autoinducer [22]; and LacI, the *lac* repressor, binds to the P_L_lac01 promoters controlling the expression of LuxR and LuxI. Except for LuxI, all proteins of this module were tagged with an 11 amino acid recognition sequence for proteases of *E*. *coli* in order to reduce their half-life [23]. Before assembling the artificial communication module (Fig 1a, *red shaded*), functionality of each single entity was tested successfully in *E*. *coli DH5α*. From these intermediate plasmids (Figure B in S1 File) the communication circuit was finally built in a modular way and examined on its own. Expression of the *luxI* and *luxR* genes was initiated by using an analogue of lactose (IPTG) that eliminates suppression of the P_L_lac01 promoters. Thus in this case, Acilhomoserine lactones, as well as LuxR should be produced from *luxI* and *luxR* respectively, which induce *aiiB* expression. These phenotypes were then tested in bioluminescence assays using the biosensor strain *E*. *coli* pSB403 [24], as shown in the supporting information (Figures C-E in S1 File). Additionally, the functionality of the assembled communication circuit (S1 and S2 Videos and Figure F in S1 File) used to establish the coupling between the repressilators in different cells was successfully tested by following the changes in the GFP expression over time. A strain transformed with the communication circuit alone was tested in the absence or in the presence of IPTG. On one hand, cells transformed with the communication circuit alone were not able to express GFP as a consequence of the LacI repression (S1 Video). On the other hand, when cells were grown in the presence of 1.5 mM IPTG the inhibition caused by LacI is release and all cells expressed GFP (S2 Video).

### Dynamics: Clustering and synchronization

**Fig 2:**
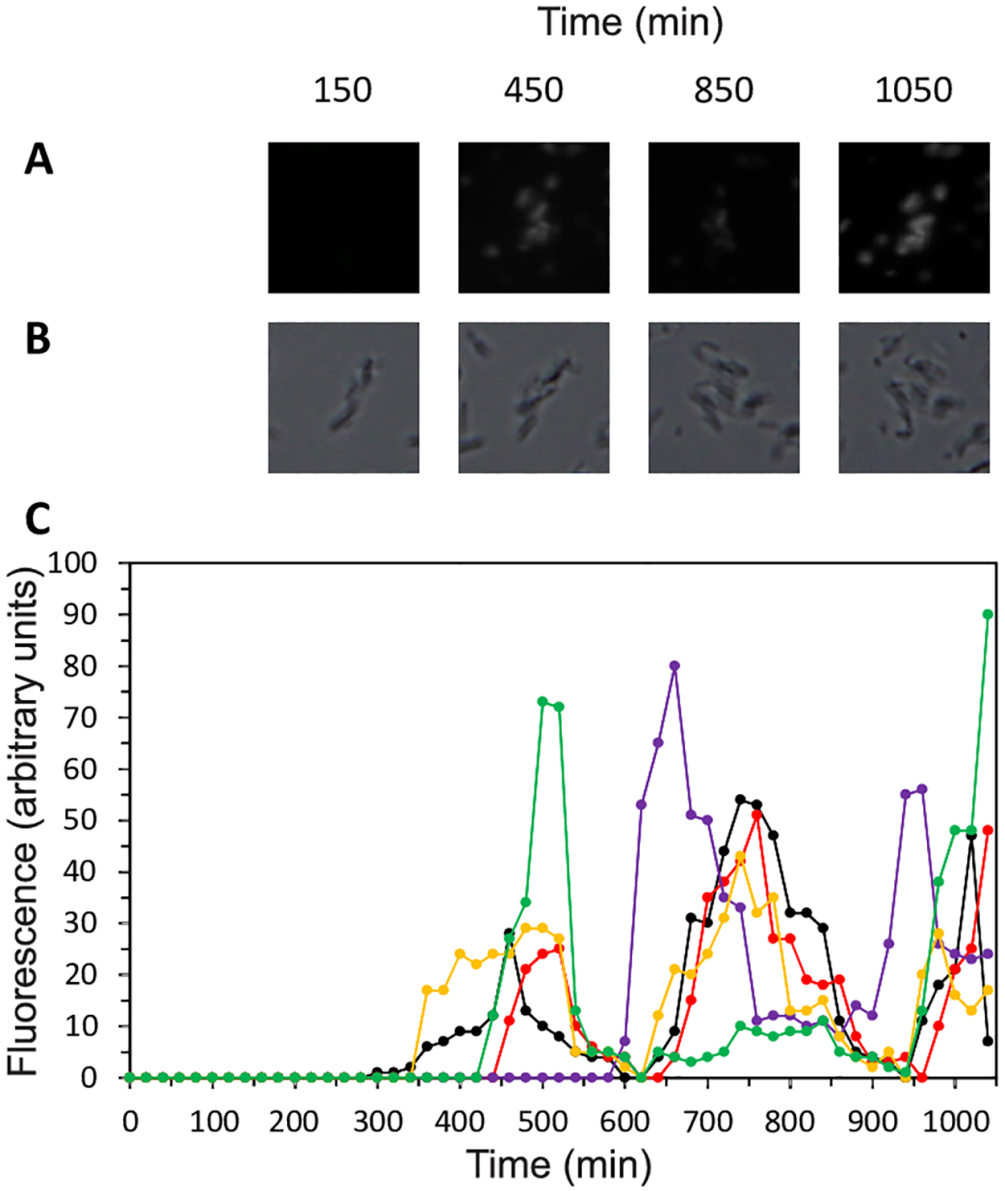
Examples of clusters of *E*. *coli* cells showing oscillatory behaviour. The *gfp* expression in clusters of cells transformed with both the repressilator and the communication plasmid was followed in a time-lapse microscope for 1080 min (S3 Video). Snapshots of five growing clusters of cells were taken periodically both in fluorescence (A) and bright-field (B). Each coloured line represents a different cluster of cells (C). Pictures in (A) and (B) correspond to the representative cluster of cells plotted as a green line in (C). Images were acquired every 20 min and the fluorescence intensity of each cluster of cells was determined using the ImageJ software.

**Fig 3:**
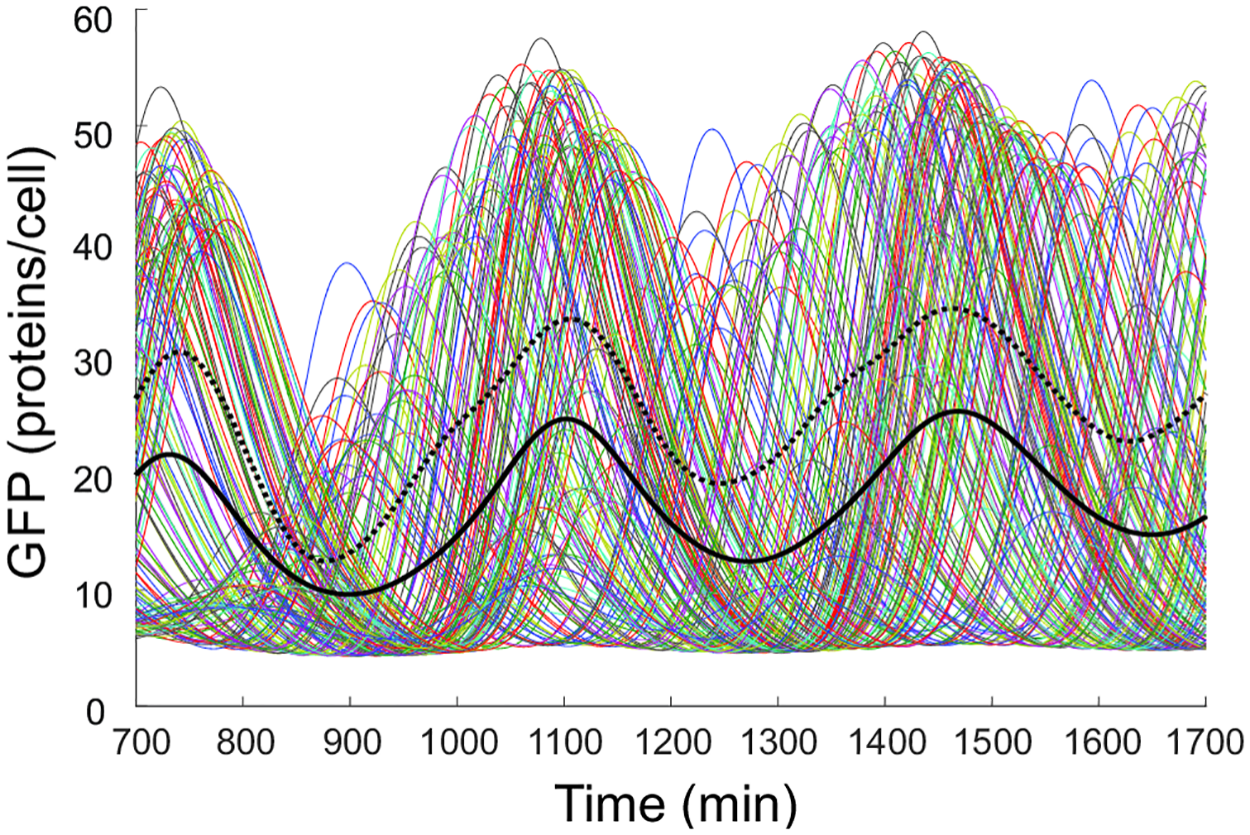
Model prediction for typical temporal oscillations of GFP in different cells. Various colours denote various cells. Black solid line represents arithmetic mean of the modelled 200 cells. Black dashed line represents weighted arithmetic mean.

In the next step, we combined the artificial communication module with the repressilator in order to study synchronization properties of a network of coupled oscillators. Bacteria, co-transformed with both, repressilator and the artificial communication module were grown in LB medium until OD 4.0 was reached. A proper amount of cells was harvested and elute in semi-solid LB medium (0.5% agar) in. order to have an OD_600nm_ = 0.2. A total of 400 μl of this culture was transferred to a μ-Dish (35 mm, high) from Ibidi (Germany). This experimental designed guaranteed that, after a transient period of about 20 min, the cells are fixed in an almost solid medium enabling the monitoring of the same cellular population over time. In distinct areas of the well, we observed clusters of cells oscillating with periods around 400 min (Fig 2 and S3 Video), a value about 2–3 times larger than that of the uncoupled repressilator (160 ± 40 min [6]). Theoretical analyses clarified that this increase in the oscillation period resulted from the coupling within the system (see below). The clustering indicates that due to the diffusing AI molecules, repressilators in different cells can “communicate” with each other, allowing global response of the investigated system. However, despite the apparent coupling within the system we still observed substantial variability: whereas in local clusters of various sizes oscillations were phase-synchronized (Figs 2 and 3), they were phase-shifted between the spatially separated clusters. Additionally, the number of oscillators populating one cluster varies over time (Fig 2b). These results indicate that even though the oscillators within one cluster oscillated in synchrony, complete synchronization of the entire cellular population of the well (all clusters of cells at the same time) was not observed under manageable observation times (up to ~1080 min). After this period of time, it was not possible to detect GFP anymore, which could be explained by the technical instability of the artificial biological circuit. In fact, it is known that the repressilator circuit can become instable over time [6, 15]

### Quantitative modeling

To quantitatively describe the mechanism of cellular clustering, as observed in the experiments, we developed a theoretical model for the investigated system (Text and Equations in S1 File). In order to model the dynamics of gene expression in the cellular population, we kept track of the temporal evolution of mRNA and protein concentrations in every cell in a network of 500 constituents. The behaviour of the system is described through ordinary differential equations in the standard way following a previously described model for a similar system [15]. According to standard approximations in quorum sensing modelling, variations in cell density were ignored and a uniform AI concentration throughout the whole culture was assumed [14, 15, 25].

In the hypothetical case, when AI → 0, the system consists of a population of isolated limit cycle oscillators—the repressilators. Under these conditions, the simulation predicts a period of approximately 270 min, which is greater than the 160 min period observed for the stand-alone repressilator. This might be due to the the additional quantity of LacI introduced by the interaction with the communication circuit. Under experimental conditions (AI ≠ 0), however, further feedback loops within the coupling module interfere with the repressilator resulting in an enlargement of the oscillation period. The model predicts a period of 325 min for individual cells in the coupled system. However, due to initial lack of synchrony at the beginning the bulk period of oscillation, which was calculated as the weighted arithmetic mean of all the cells at each time, is about 400 min, which agreed with the experimental observations. A weight was assigned to each cell as the fraction of the GFP proteins in that cell over the total amount of GFP proteins at one time. As the system synchronizes the weighted and the arithmetic means become the same. Furthermore, the simulations predict that synchronization of the whole cellular population is not achieved immediately. Instead, cell clusters of smaller size that behave in a synchronous manner are initially formed. We did not include substrate proteolytic degradation in our model as it was not included on García-Ojalvo`s model [15]. However, in order to see the significance of such degradation in our model, we carried out a simulation including this degradation. Our results showed no significant effects on the synchronization time.

**Fig 4:**
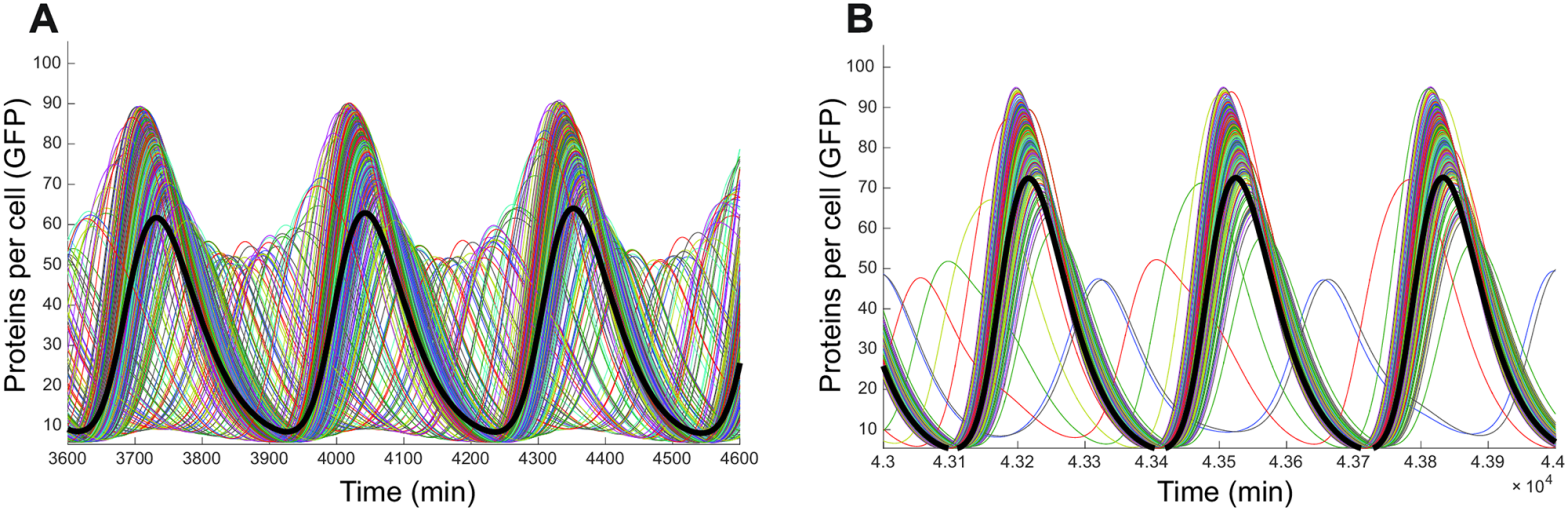
Temporal evolution of GFP in 500 cells. **(A)** Oscillations after 2 days. **(B)** Oscillations after 30 days.

Experimental observations coincide with the theoretical prediction that cells oscillate synchronously in clusters but that oscillations of different cell clusters are shifted out-of-phase. It might now be speculated that synchronization of the whole cellular population could not be observed experimentally due to the relatively long period of oscillations, which render 2–3 oscillations within the length of a performable experiment. Besides, it is known that many ensembles of coupled oscillators exhibit phase dispersion rather than a synchronized state [21,26]. In order to determine whether the population of coupled repressilators is able to self-synchronize, and which the conditions for this collective phenomenon are, we carried out simulations of the behaviour of the population over a longer time-scale finding that after approximately 2 days the system begins to display a clear but not complete synchronization among the cells (Fig 4).

**Fig 5:**
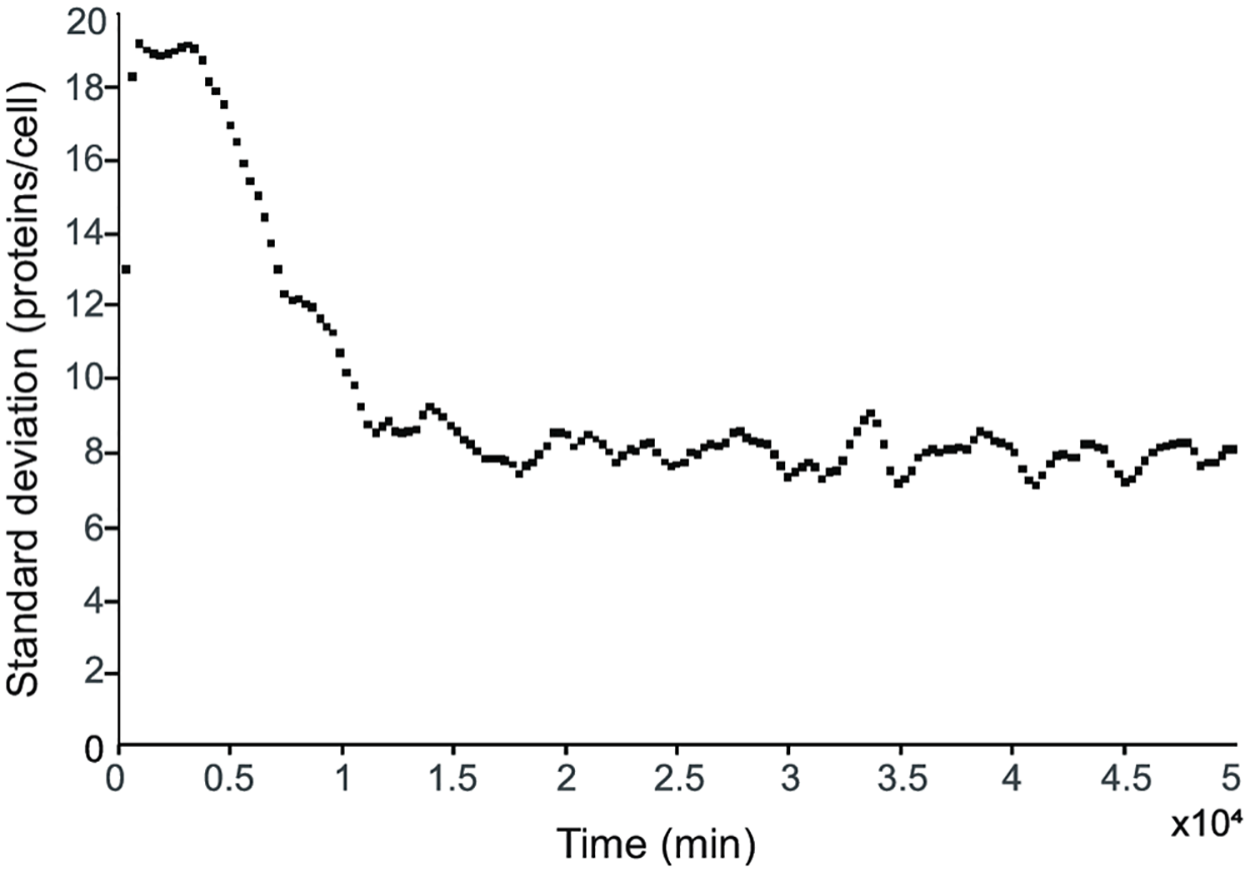
Cellular synchronization. Standard deviation of GFP proteins per cell indicates how the system evolves to a synchronized state. SD is calculated for all cells in a 5 h interval basis.

High-unsynchronized behaviour reflected by the standard deviation was detected at early times (Fig 5). Nevertheless, standard deviation becomes smaller as the cells synchronize and reaches an almost steady value. Even though the standard deviation presents large values, we believe this is due to the presence of noise incorporated in the model. System synchronization of the entire cellular population of the well was found after approximately 16000 min i.e. 11 days. The period of oscillations evolved to the period of individual cells as the system synchronizes causing it to shift from 400 min to 325 min. Here, we were able to reproduce the experimental observations and predict the behaviour of the system at times much longer than those allowed in experimental conditions. Moreover, a sensitivity analysis over the different parameters of the model showed that parameters affecting the half-life time of the proteins and the transcription rate could be used as control parameters of the system. Therefore, modifications of the protease recognition sequence and the promoters used could allow for practical tuning of the system.

### Perspectives and outlook

Synchronization is one of the fundamental nonlinear phenomena [27], shown to be inevitable for proper functioning of various biological systems, i.e. the circadian clocks in the hypothalamic suprachiasmatic nucleus [28,29] or the formation of somites in the course of vertebrate segmentation [30]. Thus, it is inevitable to ask the question: How do the underlying autonomous and intrinsically diverse oscillators manage to function in a coherent oscillatory state? We have shown here that quorum sensing can serve as a coupling mechanism for autonomous genetic clocks, establishing a well-defined test bed to study synchronization properties in natural systems. The two independent self-sustained synthetic units described here are able to autonomously oscillate in individual cells in order to produce bulk oscillations. The independence of the presented system from special microfluidic chambers is what makes it different from synthetic systems reported until now [17,18]. Our data suggest that the presented system experienced a phase transition to synchronization. This behavior is similar to that found in other models of coupled oscillators such as the Kuramoto model [31]. Due to the modularity of the presented system, it could serve additionally as a basis to construct more complex populations of synthetic units or for the construction of cooperative and competitive synthetic microbial systems [32,33]. Moreover, it can be used to study the underlying principles of various biological phenomena, such as cellular differentiation for example, for which limitations exist in the natural environment.

## Methods

### Network strains and growth conditions

The artificial cell-cell communication module (Fig 1b), plasmid pCCB9-P_luxbox_-*luxI*_is_-*aiiB*_is_-*gfp*_is_-P_Llac01_-*luxR*_is_-*LuxI* (accession number: KM219836), was built from different modules, as shown in Fig B in S1 File. Coding regions of LacI, LuxR, LuxI, AiiB, GFP, and the instability tag, the *lacI* repressor and the activating *luxbox* promoter, as well as the transcription termination signal, were all cloned by polymerase chain reaction (PCR) and verified by sequencing. Cloning primers added to each building brick a *Xba*I and/or a *Spe*I restriction site at the 5’ end and *Nhe*I and *Xho*I restriction sites at the 3’ end. In multiple cloning steps, single artificial genes comprising of a promoter, the protein-coding region and the transcriptional terminator were assembled and cloned into a minimal vector (pCCB) containing a ColE1 origin of replication and a Kanamycin-resistance gene. Every cloning step was verified by sequencing. Each constructed plasmid (pCCB1-P_Llac01_-*luxR*_is_, pCCB2-P_luxbox_-*gfp*_is_-P_Llac01_-*luxR*_is_, pCCB3-P_luxbox_-*gfp*_is_-P_Llac01_-*luxR*_is_-*LuxI*, pCCB7-P_luxbox_-*aiiB*_is_-*gfp*_is_-P_Llac01_-*luxR*_is_-*LuxI* and pCCB9-P_luxbox_-*luxI*_is_-*aiiB*_is_-*gfp*_is_-P_Llac01_-*luxR*_is_-*LuxI*) was used to transform *E*. *coli* DH5α. The strains were grown at 37°C in Louria-Bertani media (Tryptone 10g/L, yeast extract 5g/L and NaCl 10g/L, pH 7.5) with 30μg/mL kanamycin. After verification of the desired functionality of the single artificial genes, the Communication Module (pCCB9) was successively assembled. pCCB9 was co-transformed with the repressilator (Plasmid containing the repressilator was kindly provided by Michael B. Elowitz) in *E*. *coli* DH5α. Bacteria, co-transformed with both, repressilator and the artificial cell-to-cell communication module were also grown at 37°C (OD_600nm_ = 4) in LB media with Kanamycin 30 μg/ml and Ampiciline 50 μg/ml. These cells were harvest by centrifugation, and pellet was eluted in semisolid LB (0.5% agar) for microscopy (with adjustment of the OD600 to 0.2). For controls experiment, cells transformed with the the artificial cell-to-cell communication module alone were grown under the same conditions described before, with the exception that cells were resuspended in 2mL LB containing Kanamycin 30 μg/ml (control 1) and LB containing Kanamycin and IPTG 1.5 mM (control 2). In addition, the functionality of the repressilator module alone was also tested and oscillating periods between the range reported by Elowitz and Leibler (160 ± 40 min) were observed.

### Microscopy

Life imaging of cells expressing GFP was performed using a time-lapse Keyence fluorescence microscope (BZ-9000 series Generation II [Biorevo]) with a 480-nm (Ex: 465–495; DM 505; BA 515–555) filter. A volume of 400μl of the culture above described was transferred to a μ-Dish (35 mm, high) from Ibidi (Germany). This μ-Dish was left untouched enabling the monitoring of the same cell population over time. After the cells had settled (20 min), images were acquired with 30X magnification every 20 min after exposure times of 0.005 s in bright field and 0.25 s in epifluorescence through a period of 1080min. Under these conditions, the autofluorescence was negligible. The images obtained at both channels were subsequently analysed using Keyence Analysis and ImageJ software in order to assemble the movies and quantify fluorescence intensities, respectively. At each time point, the position and size (pixel region) of each cluster of cells was identified on the bright-field image. Then the fluorescence intensity data was averaged over the pre-determined pixel region in the corresponding location on the fluorescence image. Oscillation periods were estimated by the distribution of peak-to-peak intervals. At least three biological replicates of each experiment were performed.

## Supporting information

S1 Video The functionality of the assembled communication circuit was tested by following the GFP expression over time in cells transformed with the communication circuit alone. Data shows that cells are unable to express *gfp* over the curse of the experiment due to the LacI repression. Images at 30x magnification were acquired during 1080 min. At least three biological replicates of each experiment were performed.
(AVI)

S2 Video The functionality of the assembled communication circuit was tested in the presence of IPTG. Data shows that the expression of *gfp* was initiated by an analogue of lactose (IPTG) that eliminates suppression of the P_L_lac01 promoters. Images at 30x magnification were acquired during 1080 min. At least three biological replicates of each experiment were performed.
(AVI)

S3 Video Synchronization of repressilators in clusters of various sizes in a population of *E*. *coli*. The number of cells within clusters varies over time, because cells change the phase of oscillations in the initial, clustering stage. Images at 30x magnification were acquired during 1080 min. At least three biological replicates of each experiment were performed.
(AVI)

S1 File Supporting information file containing a description of our deterministic mathematical model (Text and Equations in S1 file) and six supporting figures. Figure A: Network of coupled repressilators without lactonase; Figure B: The plasmids pCCB1 and pCCB2 were designed with restriction sites flanking the genes of interest; Figure C: Acyl-HSL synthase activity assays; Figure D: Induction of bioluminescence via repressilator; Figure: AiiB lactonase activity assays by inhibition of bioluminescence and Figure F: Synthetic communication module.
(DOCX)
