# Supplementary material for: A synthetic multi-cellular network of coupled self-sustained oscillators
Source: PLoS One. 2017 Jun 29;12(6):e0180155. doi: 10.1371/journal.pone.0180155 (PMC5491139; doi:10.1371/journal.pone.0180155)
Supplement: S1 File — Figure A: Network of coupled repressilators without lactonase; Figure B: The plasmids pCCB1 and pCCB2 were designed with restriction sites flanking the genes of interest; Figure C: Acyl-HSL synthase activity assays; Figure D: Induction of bioluminescence via repressilator; Figure: AiiB lactonase activity assays by inhibition of bioluminescence and Figure F: Synthetic communication module. (DOCX) [file pone.0180155.s004.docx]

**Supporting information**

**Text and Equations**

The modular synthetic network presented in this study resembles the model proposed by Garcia-Ojalvo *et al.* (2004) to study theoretically synchronization of coupled genetic oscillators. However, the proposed model differs from those previously reported due to the addition of the lactonase, AiiB, and the inclusion of the dynamics of the LuxR-AI complex by expressing the LuxR protein under control of the P_L_lac01 promoter. These modifications are reflected in the deterministic mathematical model, which is described in detail in the following.

The processes of creation and degradation govern the concentration of the mRNA associated with the repressilator module. Taking into account that expression of each gene is subject to repression from the protein product of one of the other genes, the generation term takes the form of a Hill-type equation. The additional copy under the control of LuxR-AI activation also affects transcription of *lacI* in the system. As a convention mRNA are represented by small letters and their corresponding proteins in capital letters.

$$\frac{dc_{i}}{dt}=-l_{i}+\frac{V^{max}}{1+{T_{i}}^{n}}\left( A \right); \frac{dt_{i}}{dt}=-t_{i}+\frac{V^{max}}{1+{{LA}_{i}}^{n}}\left( B \right); \frac{d{la}_{i}}{dt}=-{la}_{i}+\frac{V^{max}}{1+{C_{i}}^{n}}+\frac{kS_{i}}{1+S_{i}} (C)$$

In this case, *c_i_, t_i_* and *la_i_* correspond to *cI, tetR* and *lacI*, respectively. The LuxR-AI is denoted as S_i_. *n* is the Hill coefficient and *k* is the maximal transcription rate when AI is saturated.

Similarly, equations for the rest of the mRNAs in the system are given by

$$\frac{dg_{i}}{dt}=-g_{i}+\frac{{V_{max}^{act}S}_{i}}{1+S_{i}}\left( D \right); \frac{d{lr}_{i}}{dt}=-{lr}_{i}+\frac{V_{max}^{tr}}{1+{LA}_{i}^{n}}\left( E \right)$$

$$\frac{d{lx}_{i}}{dt}=-{lx}_{i}+\frac{V_{max}^{tr}}{1+{LA}_{i}^{n}}\left( F \right); \frac{d{ab}_{i}}{dt}=-{ab}_{i}+\frac{V_{max}^{act}S_{i}}{1+S_{i}}(G)$$

using Michaelis-Menten kinetics for the GFP (*g_i_*) and AiiB (*ab_i_*) mRNAs and Hill-type equations for the LuxR (*lr_i_*) and LuxI (*lx_i_*) mRNAs. Given that equations D and G and equations B, E and F have the same form it is possible to assume that the kinetics of the species described by each group of equations are the same and thus, only the kinetics of GFP and TetR were modelled.

Protein kinetics were all modelled according to the following equation

$$\frac{dC_{i}}{dt}=\beta\left( c_{i}-C_{i} \right) (H)$$

where $\beta$ represent the ratio between translation rate and protein degradation. The concentration of AI is affected by degradation processes both in the cell and mediated by the lactonase, synthesis from LuxI and simple diffusion across the cell membrane.

$$\frac{dAI_{i}}{dt}=-k_{d}AI_{i}+k_{1}T_{i}-D\left( AI_{i}-AI_{e} \right)-\frac{V_{max}^{deg}{AI}_{i}}{1+{AI}_{i}}\left( I \right)$$

*k_d_* is the degradation constant in the cell, *k_1_* is the synthesis constant of LuxI, $\eta$ is the simple diffusion coefficient and *k_l_* the degradation constant of the lactonase. The synthesis term depends on the concentration of TetR, because its concentration follows the same kinetic as LuxI’s and is replacing LuxI in the model, as mentioned before.

The LuxR-AI complex concentration is modelled assuming that the creation and destruction processes of this model are faster than the other process and is thus assumed to be dependent at every time on the concentration of AI and LuxR in the cell. This dependence is modelled with a first order constant, *k_2_*.

$$S_{i}=k_{2}AI_{i}LR_{i} (J)$$

Finally, the concentration of the AI in the culture media is dependent both on degradation and diffusion from and to each cell. Following standard quorum sensing modelling assumptions, the external AI concentration is assumed to be constant throughout the culture and is unaffected by variations in cellular density.

$$\frac{dAI_{e}}{dt}=-k_{deg}AI_{i}+D_{ext}\sum_{j=1}^{N} \left( AI_{j}-AI_{e} \right)\left( K \right)$$

Additionally, quasi-steady-state is assumed rendering the following simplification of equation K.

$$AI_{e}=\frac{D_{diff,ext}\bar{AI}}{k_{deg}+D_{diff,ext}} \approx Q\bar{AI} (L)$$

$\bar{AI}$ represents the mean concentration of AI inside each individual cell. A parameter Q is defined as a ratio between the diffusion coefficient and the degradation rate. Q is directly related to cell density and can thus be used to model cells in different conditions. For example, isolated cells ($Q\to0)$ or clustered cells ($Q\to1)$.

The model is dimensionless, measuring time in units of the mRNA lifetime (assumed equal for all genes of the repressilator, t_1/2_=4 min) and the protein levels in units of their Michaelis constant. We use the following relevant experimentally scaled parameters in our simulations: $\alpha=300$, *k*=15, $\beta=0.1$, *k_d_*=1, *k_1_*=0.01, $\eta$=4, *k_l_*=0.8, h=2 and *k_2_=*0.001. The model proved to be independent of the initial conditions and always evolved to the same oscillatory behaviour.

**Figure A**

**
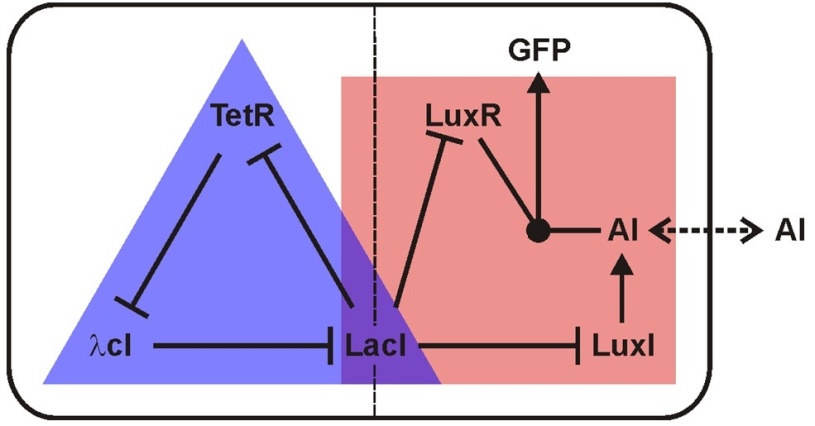

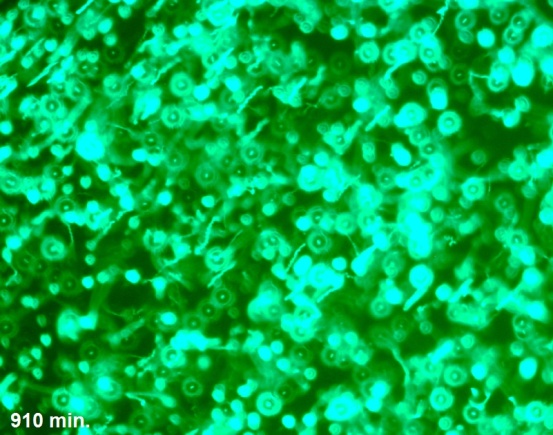
**

**Figure A. Network of coupled repressilators without lactonase.** **(Left panel)** Schematic representation of the repressilator and the communication module. **(Right panel)** Due to accumulation of AI molecules in the media, it is not possible to follow synchronized oscillations of the whole population over time. Instead, the population of coupled repressilators stays at constant expression of GFP as shown in the snapshot.

**Figure B**

**pCCB1**

**pCCB2**

**pCCB3**

***Luxbox promotor***

**T**

***Luxbox promotor***

**T**

***aiiB***

***lacI***

**pCCB7**

**pCCB9**

**Figure B.** **The plasmids pCCB1 and pCCB2 were designed with restriction sites flanking the genes of interest.** The *P_L_lac01:luxI:T* gene was released from plasmid pCCB1 using restriction enzymes and ligated into the pCCB2 plasmid vector to build the plasmid pCCB3. Alike the plasmid pCCB4 was digested and the released gene was ligated into the plasmid pCCB3 to build the plasmid pCCB7. Finally, the plasmid pCCB9 carries all the genes described in the model with the exception of those from the repressilator network. All plasmids carried the kanamycin resistance gene.

**Figure C**

**Figure C. Acyl-HSL synthase activity assays. Assay of bioluminescence**. **(A)** *E. coli* pCCB1 (*P_L_lac01:luxI:T*) and *E. coli* pCCB5 (*P_L_lac01:luxI_IS_:T*) are able to stimulate bioluminescence in the biosensor strain pSB403 (BS). For both strains, the genes are expressed only when the strains were grown on plates with IPTG. The AI molecule is able to diffuse in the media to stimulate the biosensor strain. The bioluminescence induced by the strain *E. coli* pCCB5 is less pronounced than *E. coli* pCCB1 since *E. coli* pCCB5 carries the *luxI*_IS_ gene with an upstream attenuation signal for ribosomes. **(B)** Left plate: Positive control. *Pseudomonas putida* was grown beside the biosensor strain on a plate without IPTG. *P. putida* has a Quorum Sensing system able to produce the AI molecule itself. Right plate: Negative control. The strain *E. coli* pCCB1 was grown beside the biosensor strain on a plate without IPTG. The bioluminescence was detected during 2 min of exposure in the dark. Pictures were taken with an Ultra high sensitive CCD Kamera (Image Intensifier Controller Hamamat Photon Counting Acquisition) and analyzed using the software Acuacosmos. All the experiments were performed using standard size (100 x 15 mm) petri dishes.

**Figure D**

**BS**

**pCCB3/**

**repressilator**

**Figure D. Induction of bioluminescence via repressilator**. Assay of bioluminescence. *E. coli* co-transformed with both pCCB3 and the repressilator-carrying plasmid is able to stimulate bioluminescence in the biosensor strain PSB403 when these strains are grown on plates without IPTG. The repressilator network can reduce the LacI concentration inside the cell in each cycle. The lack of the repressor LacI enables the activation of promoter P_L_lac01 on plates without IPTG. The AI molecule is able to diffuse in the media and to stimulate the biosensor strain. The bioluminescence was detected during 2 min. of exposure in the dark. Pictures were taken and analyzed as described before. All the experiments were performed using standard size (100 x 15 mm) petri dishes.

**Figure E**

**Figure E.** **AiiB lactonase activity assays by inhibition of bioluminescence**. *E. coli* pCCB1 produces stable AI only when it is grown with IPTG. This AI is able to diffuse in the media and then it can stimulate the biosensor strain. **(Upper panel)** *E. coli* pCCB7 is able to produce AiiB (lactonase). This strain was grown between the strains *E. coli-*pCCB1 and the biosensor (BS). Bioluminescence could be detected only in that region where the AI molecule is able to diffuse without being hydrolyzed by the lactonase AiiB (white arrow). **(Lower panel)** Control assay. *E. coli-*pCCB2 was grown between the strains *E. coli-*pCCB1 and BS. *E. coli-*pCCB2 did not produce AiiB. Bioluminescence detected at the entire flank indicates that there was not mechanical inhibition for the diffusion of AI by the central *E. coli* strains. Bioluminescence was detected during 2 min of exposure in the dark. All the experiments were performed using standard size (100 x 15 mm) petri dishes.

**Figure F**

**Figure F.** **Synthetic communication module**: the P_L_lac01 promoter is able to stimulate the luxR and luxI expression. luxI encodes an AI synthase. The LuxR- AI complex works as a transcription factor stimulating the transcription of genes under control of the luxbox promoter. AiiB is a lactonase which in turn degrades the AI.
